# Acute and Short-Term Autonomic and Hemodynamic Responses to Transcranial Direct Current Stimulation in Patients With Resistant Hypertension

**DOI:** 10.3389/fcvm.2022.853427

**Published:** 2022-03-11

**Authors:** Bruno Rodrigues, Catarina A. Barboza, Eliezer G. Moura, Gabriela Ministro, Silvia E. Ferreira-Melo, Javier B. Castaño, Wilton M. S. Nunes, Cristiano Mostarda, Antonio Coca, Lauro C. Vianna, Heitor Moreno-Junior

**Affiliations:** ^1^Laboratory of Cardiovascular Investigation & Exercise, School of Physical Education, University of Campinas (UNICAMP), Campinas, Brazil; ^2^Laboratory of Cardiovascular Pharmacology & Hypertension, School of Medical Sciences, University of Campinas (UNICAMP), Campinas, Brazil; ^3^Physical Education Department, Federal University of Maranhão (UFMA), São Luís, Brazil; ^4^Hypertension and Vascular Risk Unit, Hospital Clínic, University of Barcelona, Barcelona, Spain; ^5^NeuroV̇ASQ̇ - Integrative Physiology Laboratory, Faculty of Physical Education, University of Brasília, Brasília, Brazil

**Keywords:** transcranial direct current stimulation (tDCS), resistant hypertension, blood pressure, autonomic nervous system, biochemical analyses

## Abstract

Previously, we demonstrated that acute transcranial direct current stimulation (tDCS) reduced blood pressure (BP) and improved autonomic modulation in hypertensives. We hypothesized that acute and short-term tDCS intervention can promote similar benefits in resistant hypertensive patients (RHT). We assessed the impact of one (acute intervention) and ten (short-term intervention) tDCS or SHAM (20 min, each) sessions on BP, pulse interval (PI) and systolic blood pressure variabilities, humoral mechanisms associated with BP regulation, and cytokines levels. True RHT subjects (*n* = 13) were randomly submitted to one and ten SHAM and tDCS crossing sessions (1 week of “washout”). Hemodynamic (Finometer^®^, Beatscope), office BP, and autonomic variables (accessed through spectral analysis of the pulse-to-pulse BP signal, in the time and frequency domain – Fast Fourrier Transform) were measured at baseline and after the short-term intervention. 24 h-ambulatory BP monitoring was measured after acute and short-term protocols. Acute intervention: tDCS reduced BP, cardiac output, and increase high-frequency band of PI (vagal modulation to the heart). Short-term protocol: tDCS did not change BP and cardiac output parameters. In contrast, central systolic BP (−12%), augmentation index (−31%), and pulse wave velocity (34%) were decreased by the short-term tDCS when compared to SHAM. These positive results were accompanied by a reduction in the low-frequency band (−37%) and an increase of the high-frequency band of PI (+62%) compared to SHAM. These findings collectively indicate that short-term tDCS concomitantly improves resting cardiac autonomic control and pulse wave behavior and reduces central BP in RHT patients, https://ensaiosclinicos.gov.br/rg/RBR-8n7c9p.

## Introduction

Resistant hypertension (RHT) is a clinical condition defined as above-goal elevated blood pressure in a patient, despite optimal use of ≥3 antihypertensive classes of drugs (which includes a diuretic agent), administered at maximally tolerated doses (1). Patients with RHT are at higher risk for target organ damage, morbidity, and mortality despite ongoing antihypertensive drug therapy (2). Elevated sympathetic activation and impaired renin-angiotensin-aldosterone components have been established in the early stages of hypertension, suggesting that neurohormonal dysregulation may play a pivotal role in its etiology (3), the progression of hypertension, and subsequent end-organ damage, such as raised arterial stiffness (4). However, despite standard drug therapy, sympathetic nerve activity remains high in RHT patients making the autonomic nervous system a primary target in the treatment of RHT (5, 6). As such, newer interventional strategies are needed to enhance the autonomic neural control of the cardiovascular system and potentially improve the clinical prognosis for RHT patients (7). Given the significant financial costs associated with developing novel pharmaceutical drugs, there is increasing interest in non-pharmacological alternatives.

Technological devices were developed to treat RHT by inhibiting sympathetic activity, including activation of the carotid baroreceptors using electrical stimuli (8) and selective renal sympathetic denervation (9), providing relevant results of blood pressure lowering (7–11). However, these device-based approaches require an invasive surgical procedure, and as such, this may limit its viability for RHT therapy. Given these considerations, noninvasive brain electrical stimulation has been tested, with beneficial cardiovascular outcomes for healthy subjects and hypertension patients (12–14). Briefly, transcranial direct current stimulation (tDCS) (13) is a non-pharmacological and noninvasive intervention for treating or preventing depressive episodes, epilepsy crisis, stroke motor sequela, migraine, fibromyalgia, chronic pain control (12), and other neuropsychiatric disorders (15, 16). Low-intensity tDCS in humans appears to be safe. Of note, no serious adverse events have been reported in more than 18,000 sessions administered to healthy subjects or neurological and psychiatric patients (16). Two other favorable characteristics are the relatively low cost and the straightforward operation of the equipment. Although the current literature indicates a potential role of tDCS on blood pressure control via an increase of vagal modulation (17–20), to our knowledge, no studies have specifically examined the effects of tDCS on autonomic and cardiovascular responses as the primary outcome in patients with RHT.

Since those studies using functional magnetic resonance imaging to assess the connection of the M1 area and autonomic nervous system were not found, it is already known that cortical motor areas, including M1, project directly to the reticular formation regions and the spinal cord (21–23), and motor network on the adrenal medulla is mediated by corticospinal and corticobulbo-spinal pathways. Classical physiologic studies demonstrated that stimulation of M1, primary somatic sensory cortex (S1), and dorsal premotor areas evoked changes in blood pressure (24, 25).

We have previously demonstrated that an acute session of tDCS in M1 area tDCS promoted positive adjustments on cardiac autonomic control and reduced 24-hs blood pressure values of non-RHT patients (14). Here, we hypothesized that an acute session of tDCS could also reduce sympathetic modulation, increase vagal modulation, and decrease blood pressure in RHT patients. Furthermore, we believed that ten consecutive sessions of tDCS could exert a positive summation effect on this regulatory mechanism and blood pressure values.

Given this background, the purpose of the present clinical trial was to rigorously assess the effects of one (i.e., acute) and ten (i.e., short-term) tDCS sessions on the blood pressure levels and neurohumoral mechanisms in RHT subjects. Considering that vagal activation seems to suppress pro-inflammatory cytokines production (26–28), we also measured inflammatory cytokines (interleukin-10 and TNF-α) and circulating hormones (cortisol and noradrenaline).

## Materials and Methods

### Patients

Thirteen resistant hypertensive volunteers from the Outpatient Hypertension Clinic of the University of Campinas (UNICAMP, Campinas, Brazil) were screened for this study. This protocol was approved by the Ethical in Research Committee of the School of Medical Sciences, University of Campinas (Campinas, Brazil) and performed following the Declaration of Helsinki. All participants signed a written consent form before being included in the study (approval no.2.681.083/CAAE: 86618317.0.0000.5404). This study was registered at the Brazilian Registry of Clinical Trials (ReBEC) under Register Number: RBR-8n7c9p.stem.

Patients were included after at least a 6-month screening protocol for diagnosing RHT and check the adherence to pharmacological and non-pharmacological therapy. Two ambulatory blood pressure monitoring (ABPM) were performed to exclude white-coat hypertension, and pill counts assessed lack of adherence. Patients with an adherence rate below 80% of the prescribed medication were excluded from our sample. We tested patients for: renal artery stenosis, pheochromocytoma, primary hyperaldosteronism (aldosterone renin ratio – ARR > 20 ng/dL per ng/mL per hour), Cushing syndrome (by assessing cortisol and adrenocorticotropic hormone levels), and obstructive sleep apnea (classified as “high risk” according to Berlin questionnaire), to exclude secondary causes of hypertension. Thus, after patients were screened for possible causes of pseudo-RH, they were defined as those using ≥3 antihypertensive agents of different classes, including a diuretic (5).

Other exclusion criteria were: clinically evident coronary artery or cerebrovascular diseases, significant impaired renal or liver function, myocardial infarction or peripheral vascular disease, use of pacemakers or other implanted electronic devices, and depression (≥17 points on Hamilton's scale) (29).

### Study Design

**Figure 1:**
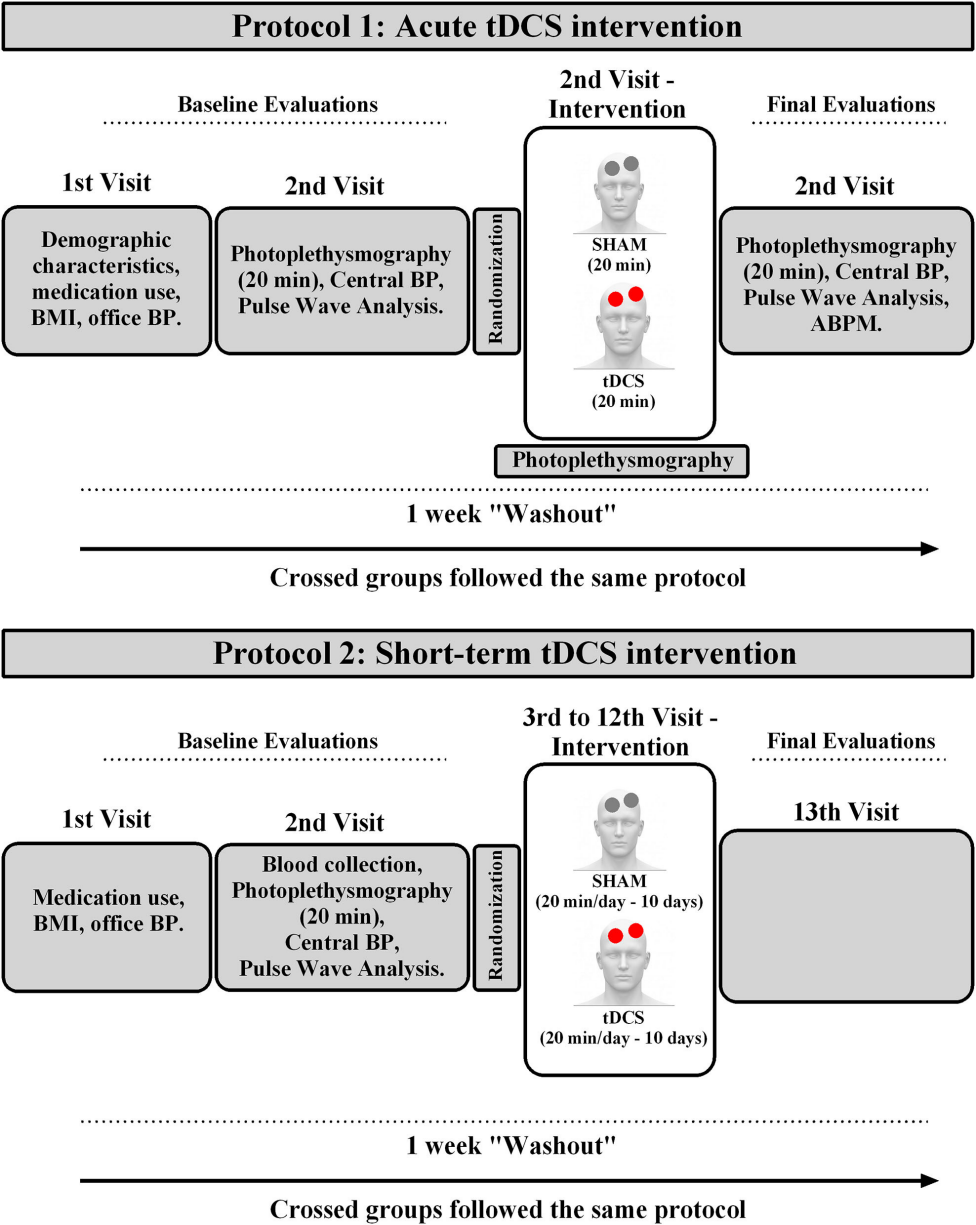
Experimental design. BMI, body mass index; ABPM, ambulatory blood pressure measurement.

This study had a double-blind, randomized crossover with a placebo (SHAM) design. Participants were involved in the study for two protocols, as showed in Figure 1.

#### Protocol-1: Acute Intervention

The participants were interviewed for their demographic characteristics and medication use on the first visit, assessed for body weight, height, and office blood pressure. On the second visit, applanation tonometry was performed to assess noninvasive central hemodynamic variables (SphygmoCor CPV system, AtCor Medical, USA). After this procedure, subjects were assessed to 20 min of photoplethysmography with beat-to-beat signal (Finometer®, Finapres Medical System BV, Netherlands) to evaluate hemodynamic and autonomic variables. Then, subjects participated in the first randomized stage of the protocol. An investigator not involved in the assessment, intervention, or statistical analysis conducted the randomization (1:1) of subjects and programmed the device for the tDCS or SHAM sessions. After device programming by this investigator, another researcher started the session, blinded to the type of intervention that the device was programmed. tDCS (Model DC-Stimulator Plus; NeuroConn GmbH, Germany) was performed with anodal excitation, 0.06 mA/cm^2^, in the primary motor cortex, M1 region, for 20 min. Placebo (SHAM) sessions were constituted by the electrode positions and stimulation parameters identical to that used for anodal stimulus. The excitation stopped after a ramp-up and ramp-down period of the 30s each to provide an equivalent scalp sensation. Hemodynamic and autonomic measurements were conducted during the 20 min of SHAM and tDCS stimulation. (photoplethysmography). After the acute intervention, for 20 min, beat-to-beat photoplethysmography continued being assessed. Then, central hemodynamic variables were evaluated, and 24 h-ambulatory blood pressure measurements (ABPM) were started. After 1 week of “washout,” subjects were submitted to the same procedures, crossing the type of intervention (SHAM or tDCS).

#### Protocol-2: Short-Term Intervention

After 1 week of Protocol 1, the same subjects were enrolled in thirteenth visits over 3 weeks. The participants were interviewed for their medication use on the first visit, assessed for body weight, height, and office blood pressure. After 12 h of fasting, on the second visit, blood samples were collected (8:00–9:00 h a.m.) to analyze biochemical markers. After a standardized breakfast, subjects were assessed for 20 min of photoplethysmography with a beat-to-beat signal to evaluate hemodynamic and autonomic variables. Then, applanation tonometry was performed to assess noninvasive central hemodynamic variables and pulse wave analysis.

On the third visit, subjects participated in the first randomized stage of the protocol. An investigator not involved in the assessment, treatment, or statistical analysis conducted the randomization (1:1) of subjects and programmed the device for the tDCS or SHAM sessions. After device programming by this investigator, another researcher started the session, blinded to the type of intervention that the device was programmed. tDCS and SHAM sessions were similar to the acute protocol and occurred between 8:00–12:00 h a.m. after patients took their usual antihypertensive medication. Such protocols were applied for 10 days.

Thereby, patients received tDCS or Sham interventions on consecutive days from the third to the twelfth visit. On the thirteenth visit (24 h after the last tDCS session), patients were reassessed by biochemical markers, office blood pressure, hemodynamic and autonomic variables, as well as central blood pressure and pulse wave analyses. Immediately after the recording, 24 h-ABPM was carried out. After 1 week of “washout,” participants initiated the same experimental sequence performed with the crossing of subjects, i.e., those selected for Sham stimulation in the previous stage, tDCS was performed, and vice-versa.

### Procedures

#### Office BP Measurements

Office BP was measured using a certified digital sphygmomanometer (HEM-907 XL OMRON Healthcare Inc., Bannockburn, IL, USA) by a trained health professional, according to the European Society Hypertension (ESH) 2018 guidelines (30).

#### Biochemical Analyses

Blood samples were collected by venipuncture in heparinized vacutainers after 12 hs fasting and immediately centrifuged at 4,000 rpm for 5 min to separate plasma. The TNF-α, IL-10, noradrenaline, and cortisol concentrations were determined in peripheral blood by the enzyme-linked immunosorbent assay (ELISA) technique using high detection sensitivity kits (R&D System, a biotech brand, Quantikine ELISA, Inc., Minneapolis, USA) with ranges between 15.6–1,000 pg/mL for TNF-α, and 7.8–500 pg/ml for IL-10. Intra- and inter-assay variations (%) were 4.2–5.2 and 4.6–7.4 for TNF-α, and 1.7–5.0 and 5.9–7.5 for IL-10. Noradrenaline ranges between 0.313 and 20 ng/mL, and cortisol ranged from 8.5 to 23.8 μg/dL. Intra- and inter-assay variations (%) were 5.5–8.4 for noradrenaline and 7.2–10.9 for cortisol.

#### Hemodynamic and Autonomic Indexes

With patients in a sitting position, after 15 min of rest, continuous beat-to-beat blood pressure waves were obtained by a digital photoplethysmography device (Finometer®, Finapress Medical System BV, Netherlands) for 20 min. A software program (BeatScope) used BP curves and patient data (age, sex, body mass, and stature) to calculate systolic and diastolic BP (SBP and DBP), heart rate (HR), cardiac output (CO), and peripheral vascular resistance (PVR). The waveforms were simultaneously recorded on another computer equipped to acquire and convert the biological signals AT/MCA-CODAS (DATAC Instruments Inc., Akron, Ohio, USA). The sampling frequency of signals was 1,000 Hz.

The stored data from photoplethysmography underwent a routine analysis (spectral analysis) to provide pulse interval (PI) and systolic blood pressure (SBP) variability. Although the PI variability assessment may be considered may be less accurate than measuring heart rate variability by electrocardiogram, some studies have demonstrated the agreement between heart rate variability and PI variability (31, 32).

Beat-to-beat BP was analyzed using a specialized algorithm for spectral data analysis (CardioSeries Software, version 2.4, Ribeirão Preto, SP, Brazil), which automatically detects SBP and DBP waves. Pulse interval (PI) was calculated as the difference between the cycle's start and endpoints (t1-t0). The spectral power density of the SBP and the PI range were computed using the Fast Fourier Transform and the Welch method. To set the window length was established in 5 min.

In the time domain, we analyzed: SDNN [standard deviation of normal-to-normal (NN) PI] and PI Variance (total variance of PI); RMSSD (the square root of the mean of the sum of the squares of differences between adjacent NN intervals, which represents cardiac vagal modulation of PI) and SBP Variance (variance of systolic blood pressure in short-time). The spectral bands evaluated for humans were defined as very-low-frequency (VLF: 0.007–0.04 Hz), low-frequency (LF: 0.04–0.15 Hz), high-frequency (HF: 0.15–0.4 Hz), and total power. The normalized values (nu) for the LF and HF bands were then calculated using the predefined formulae: LF (n.u.) = LF/(total power spectral density – VLF) ×100 or HF (n.u.) = HF/(total power spectral density – VLF) ×100. The ratio for the absolute values for the LF band of PI and HF band of PI (LF/HF) was also calculated as a representative of autonomic balance.

The HF component of PI variability has been related to the efferent vagal modulation. However, the interpretation of the LF component of PI is more controversial since that includes the influences of sympathetic and parasympathetic modulation (33). Also, there is evidence that the LF component of SBP variability is influenced by sympathetic modulation of vascular tone and myogenic vascular function (34). Furthermore, the assessment of blood pressure variability in very short-term (beat-to-beat) reflects the influences of central and reflex autonomic modulation, elastic properties of arteries, humoral and emotional factors (35).

#### Central Blood Pressure and Pulse Wave Assessment

Applanation tonometry was performed to assess noninvasive central hemodynamic variables and pulse wave velocity (PWV) using the SphygmoCor system (AtCor Medical, Sydney, Australia). Consecutive measurements of the carotid and femoral artery pulse waves were electrocardiogram gated. The distance between the two sites was measured on the body surface to determine aortic PWV in meters/second (m/s). The total distance between the carotid and femoral arteries was used for measurement. The average measurements throughout 8 s (9–10 cardiac cycles) were calculated after excluding extreme values (values above or below four standard deviations).

After 20 sequential waveforms were acquired and averaged, a validated generalized mathematical transfer function was used to synthesize the corresponding central aortic pressure wave (36).

The Augmentation Index (AIx), defined by the ratio between the pressure exerted by the reflected wave and the ejection wave, was evaluated (37). This index is expressed as a percentage of the Central Pulse Pressure [AIx = BP/central pulse pressure (cPP) ×100%]. Since AIx is influenced by heart rate, an index normalized for 75 bpm was used too. The patients were required to abstain from smoking and consuming alcohol or coffee 24-h before the procedure.

#### Transcranial Direct Current Stimulation (tDCS)

The primary motor cortex region (M1) was chosen as the target for tDCS and was stimulated by a constant current stimulator (Model DC-Stimulator Plus; NeuroConn GmbH, Germany). With a 5.0 ×7.0 cm electrode (35 cm^2^ area) housed in saline-soaked sponges, an anodal stimulation in the M1 region was positioned in the C3 area (using the International 10–20 EEG system) the cathode was placed in the supraorbital region. A current density of 0.06 mA/cm^2^ and an intensity of 2.0 mA were applied on the scalp during 20 min per active session. For placebo SHAM, the electrode positions and stimulation parameters were the same as those used for anodal stimulation. The stimulation stopped after a ramp-up and ramp-down period of the 30 s each to provide an equivalent scalp sensation. Thus, for SHAM stimulation, the device switched off automatically after the 30 s of stimulation. The device display remained, indicating the stimulation time, regardless of whether the active or SHAM current was provided. Previous work demonstrated that this type of blinding is effective at low stimulation intensities (38). In the first session (SHAM and tDCS), we encouraged patients to describe stimulation sensation. Afterward, we asked if the perceived sensation was similar to the previous session (14).

#### Ambulatory Blood Pressure Measurement (ABPM)

ABPM was carried out with an automatic oscillometer device (Spacelabs 90207, Spacelabs Inc), and only records with more than 85% of total measures were analyzed. Those patients who had more than 25% of incomplete measurements should retake the exam. However, no patient had to be re-examined. The device was then set to obtain four blood pressure readings per hour (one every 15 min). The parameters measured were average 24-h, daytime and nighttime of systolic BP, diastolic BP, mean BP, and pulse pressure.

### Statistical Analyses

Analyses were performed using the GraphPad Prism 6.0 software. The normality of data and homogeneity of variance were assessed using the Shapiro-Wilk and Levene tests, respectively. Mauchly's test was used to evaluate the sphericity assumption and, whenever sphericity was violated, Greenhouse-Geiser epsilon correction was used. We estimated sample size was using G^*^Power software to ANOVA (version 3.1.9.2.), based on the magnitude of the mean differences in systolic blood pressure levels among tDCS or SHAM sessions (13). The sample size was estimated to be 10 participants per group, considering an ES set at 0.45 (13), a power of 80%, and a level of significance at 5%. G^*^Power calculations are based on a nondirectional χ2-test situation. ABPM was analyzed with a 2-tailed paired *t*-test. Hemodynamic, autonomic, and biochemical parameters were analyzed by repeated-measures two-way ANOVA, followed by Bonferroni post-test. The significance level was set at *p* ≤ 0.05. Data are expressed as mean ± standard deviation. Pearson's correlation was used to analyze the associations between variables at final moment (short-term protocol) of SHAM and tDCS conditions. Considering that the LF band of PI represents, at least partly, the influence of sympathetic modulation, this parameter was chosen as an independent variable for associations with cortisol levels and central blood pressure. As vagal activation has been related to a reduction in the inflammatory profile (26–28), we chose the HF band of PI as the independent variable and tested its association with IL-10 levels. Finally, knowing that inflammatory profile can directly influence vascular morphology and modify pulse wave behavior, IL-10 levels were chosen as an independent variable in the association with AIx. The variance inflation factor (VIF) was used to check the co-linearity. The reported relationships remained while controlling for other predictor variables.

## Results

The sample consisted of 13 RHT subjects (68 ± 7 years old; 7 women). Table 1 shows the characteristics of the subjects enrolled in the present study. All subjects tolerated tDCS well, and most patients reported scalp tingling sensations that decreased in intensity over time in all SHAM and tDCS sessions. None of them reported any side effects upon request.

**Table 1:** Characteristics of patients enrolled in acute and short-term protocols.

**Resistant Hypertensive Subjects (*n* = 13)**	
**Clinical data**	
Age (yrs)	68 ± 7
Women, *n* (%)	7 (63)
Diabetes mellitus, *n* (%)	4 (36)
BMI (Kg/m^2^)	29.3 ± 9.4
Free fat mass (kg)	56 (45–73)
Fat mass (kg)	27 ± 9
Office SBP (mmHg)	148 ± 14
Office DBP (mmHg)	87 ± 13
Heart rate (bpm)	77 ± 12
**Antihypertensive drugs**	
N. of Classes	4 ± 1
Diuretics, *n* (%)	13 (100)
Espironolactone, *n* (%)	3 (27)
Beta-blockers, *n* (%)	6 (54)
ACEi and ARBs, *n* (%)	12 (92)
CCBs, *n* (%)	8 (72)

*BMI, body mass index; SBP, systolic blood pressure; DBP, diastolic blood pressure; ACEi, angiotensin-converting enzyme inhibitors; ARBs, angiotensin receptor blockers; CCBs, calcium channel blockers*.

### Protocol 1. tDCS Acutely Reduces Blood Pressure, Possibly Meditated by Positive Changes in Autonomic Modulation

Since the hemodynamic effects of tDCS in resistant hypertensive patients are not known, we tested its acute effects on blood pressure and associated variables. To begin to uncover it, we assessed the pulse moment-by-moment (plethysmography) at Baseline, During, and After tDCS or SHAM session, as observed in Table 2. tDCS lead to a reduction in the systolic, diastolic and mean blood pressure, as well as in cardiac output, and peripheral vascular resistance after stimulation as compared with Baseline, and SHAM at after moment.

**Table 2:** Acute intervention: hemodynamic assessments of resistant hypertensive patients at baseline, during, and after tDCS or SHAM conditions.

		**Baseline**	**During**	**After**	***P* Intragroup**	***P* vs. Sham - After**
**Hemodynamic variables**		
SBP (mmHg)	SHAM	130 ± 17	128 ± 13	132 ± 18	0.5471	0.0152
	TDCS	138 ± 14	121 ± 12	116 ± 10^†^^*^	0.0023	
DBP (mmHg)	SHAM	84 ± 8	87 ± 9	87 ± 6	0.6012	0.0012
	TDCS	87 ± 13	77 ± 14^*^	67 ± 11^†^^*^	0.0010	
MBP (mmHg)	SHAM	99 ± 8	101 ± 8	101 ± 8	0.6471	<0.0001
	TDCS	104 ± 10	91 ± 10^†^	83 ± 8^†^^#^^*^	0.0002	
HR (bpm)	SHAM	65 ± 10	65 ± 11	65 ± 11	0.7023	0.7587
	TDCS	66 ± 12	66 ± 10	67 ± 11	0.6987	
CO (L/min)	SHAM	6 ± 2	6 ± 2	6 ± 1	0.8795	0.0002
	TDCS	7 ± 2	5 ± 1^†^^*^	5 ± 1^†^^*^	0.0001	
PVR (dyn.s/cm^5^)	SHAM	4,536 ± 2,128	5,220 ± 2,204	5,187 ± 2,692	0.5907	0.0005
	TDCS	5,362 ± 1,854	3,320 ± 1,749	2,006 ± 1,226^†^^*^	0.0002	
**Autonomic variables**		
PI Variance (ms^2^)	SHAM	576 ± 124	627 ± 49	623 ± 48	0.7457	0.0042
	TDCS	625 ± 64	961 ± 65	1600 ± 62^†^^*^	0.0025	
RMSSD (ms)	SHAM	32 ± 16	29 ± 13	31 ± 14	0.6981	0.0033
	TDCS	29 ± 7	37 ± 10^†^	48 ± 9^†^^*^	0.0022	
LF-PI (n.u.)	SHAM	71 ± 10	71 ± 10	73 ± 9	0.5782	0.0001
	TDCS	67 ± 13	62 ± 17	26 ± 10^†^^*^	0.0001	
HF-PI (n.u.)	SHAM	29 ± 10	28 ± 10	27 ± 9	0.6201	0.0045
	TDCS	32 ± 10	47 ± 16	73 ± 9^*^^†^	0.0052	
LF/HF	SHAM	2.87 ± 1.47	2.96 ± 1.46	3.19 ± 1.68	0.5211	0.0002
	TDCS	2.60 ± 1.71	1.45 ± 1.04^†^^*^	0.45 ± 0.21^#^^†^^*^	0.0004	
SBP Variance (mmHg^2^)	SHAM	72.3 ± 12	72.6 ± 11	73.9 ± 9	0.6830	<0.0001
	TDCS	69.1 ± 9	55.2 ± 7^*^	46.1 ± 6^†^^*^	0.0001	
LF-SBP (mmHg^2^)	SHAM	15 ± 4.8	13 ± 3.9	13. ± 4.4	0.4877	0.0004
	TDCS	14 ± 4.7	9 ± 4.4^†^^*^	6.5 ± 3.6^†^^*^	0.0002	

*Data are expressed as mean ± standard deviation. Repeated measures ANOVA with Bonferroni post-hoc. SBP, systolic blood pressure; DBP, diastolic blood pressure; MBP, mean arterial pressure; HR, heart rate; CO, cardiac output; SDNN, average of the standard deviations of the normal pulse intervals every 5 min; VAR-PI, pulse interval variance; RMSSD, the root of the mean of the square of the differences between adjacent normal pulse intervals; LF-PI, low-frequency band of the pulse interval; HF-PI, the high-frequency band of the pulse interval.*

* *P < 0.05 vs. SHAM at after moment;*

† *P < 0.05 vs. Baseline in the same condition;*

# *P < 0.05 vs. During in the same condition*.

Next, we focused on autonomic modulation and investigated whether this regulatory mechanism can explain the observed effects of tDCS on blood pressure (Table 2). As expected, the SHAM protocol did not cause changes in cardiac and peripheral autonomic variables. It was possible to observe that tDCS increased the variance of pulse interval (PI Variance); the root of the mean of the square of the differences between adjacent normal RR intervals (RMSSD); the high-frequency band of pulse interval (HF-PI) if compared with the baseline evaluations and SHAM at after moment. Low-frequency band of pulse interval (LF-PI); LF/HF ratio; the variance of systolic blood pressure (SBP Variance), and low-frequency band of systolic blood pressure (LF-SBP) were reduced after tDCS stimulation compared with the baseline evaluation and SHAM at after moment.

To determine whether tDCS can extend its effects beyond 20 min post-stimulation, we evaluated 24-h blood pressure through ambulatory blood pressure monitoring (ABPM). At 24-h measurement, SBP, DBP, mean BP (MBP), and pulse pressure (PP) were reduced by tDCS. During the daytime, tDCS did not change these variables, except for PP values, which were reduced after tDCS. In the nighttime, tDCS reduced SBP, DBP, MBP, and PP (Table 3). These results indicate that acute tDCS presents positive effects on office blood pressure, possibly mediated by improved autonomic modulation, reducing cardiac and peripheral sympathetic modulation, and increasing parasympathetic regulation to the heart. The hemodynamic effects of tDCS were extended during 24-h, as evaluated by ABPM.

**Table 3:** Acute intervention: 24-hs-ambulatory blood pressure measurements (ABPM) in resistant hypertensive patients after SHAM or tDCS conditions.

	**SHAM**	**TDCS**	***P***
**24 h**			
SBP (mmHg)	137 ± 9	122 ± 10^*^	0.0039
DBP (mmHg)	90 ± 6	64 ± 8^*^	0.0041
MBP (mmHg)	96 ± 7	82 ± 9^*^	0.0002
PP (mmHg)	57 ± 7	54 ± 6^*^	0.0215
**Daytime**			
SBP (mmHg)	138 ± 7	127 ± 7	0.8182
DBP (mmHg)	91 ± 7	69 ± 6	0.1661
MBP (mmHg)	97 ± 8	89 ± 7	0.3955
PP (mmHg)	57 ± 7	53 ± 5^*^	0.0146
**Nighttime**			
SBP (mmHg)	135 ± 15	118 ± 13^*^	0.0046
DBP (mmHg)	77 ± 8	62 ± 8^*^	0.0017
MBP (mmHg)	92 ± 10	80 ± 9^*^	0.0023
PP (mmHg)	58 ± 10	54 ± 9^*^	0.0036

*Data are expressed as mean ± standard deviation. 2-tailed paired t-test. SBP, systolic blood pressure; DBP, diastolic blood pressure; MBP, mean blood pressure; PP, pulse pressure.*

* *P < 0.05 vs. SHAM*.

### Protocol 2. Short-Term (10 Sessions) tDCS Intervention Does Not Change Peripheral Blood Pressure, Despite Improving Central Blood Pressure and Autonomic Modulation

Knowing that acutely tDCS promotes blood pressure reduction and improved autonomic modulation, we tested whether short-term stimulation can promote similar benefits in these patients. To test this hypothesis, we submitted the resistant hypertensive patients to ten sessions of tDCS and SHAM stimulation (randomized, double-blinded crossover design, with the crossing occurring after 1 week of each intervention).

Despite the results observed in the acute protocol, ten sessions of tDCS did not change systolic, diastolic, mean blood pressures, pulse pressure, heart rate, cardiac output, and peripheral vascular resistance as compared with SHAM at the end of intervention (Table 4).

**Table 4:** Short-term intervention (10 sessions): hemodynamic variables at baseline and final of the tDCS or SHAM conditions.

		**SHAM**	**TDCS**	***P* Intragroup**	***P* vs. Sham - Final**
SBP (mmHg)	Baseline	133.4 ± 7.8	139.6 ± 15.5	0.3211	0.4357
	Final	134.8 ± 12.3	137.2 ± 8.2	0.2341	
DBP (mmHg)	Baseline	68.6 ± 13.3	73.1 ± 11.5	0.6540	0.4532
	Final	79.2 ± 13.6	78.2 ± 8.5	0.7421	
MBP (mmHg)	Baseline	92.4 ± 15.0	95.7 ± 16.1	0.4410	0.2653
	Final	102.8 ± 12.1	101.2 ± 9.2	0.5023	
PP (mmHg)	Baseline	58.4 ± 4.2	66.6 ± 9.9	0.1240	0.5648
	Final	55.6 ± 8.32	58.2 ± 13.2	0.2988	
HR (bpm)	Baseline	64.4 ± 6.9	58.1 ± 8.2	0.0878	0.3455
	Final	71.2 ± 13.1	69.1 ± 4.9	0.6674	
CO (L/min)	Baseline	6.3 ± 3.1	6.6 ± 2.8	0.7455	0.4892
	Final	6.4 ± 2.6	5.5 ± 3.7	0.5212	
PVR (dyn.s/cm^5^)	Baseline	11,989.2 ± 5,763.6	11,698.2 ± 4,835.7	0.7742	0.5982
	Final	12,397.3 ± 6,874.2	10,458.4 ± 5,231.8	0.0755	

*Data are expressed as mean ± standard deviation. Repeated measures ANOVA with Bonferroni post-test. SBP, systolic blood pressure; DBP, diastolic blood pressure; MBP, mean blood pressure; PP, pulse pressure; HR, heart rate; CO, cardiac output; PVR, peripheral vascular resistance*.

Regarding 24 h-ambulatory blood pressure measurements, after short-term tDCS intervention, we did not observe changes in 24 h, daytime, and nighttime of systolic, diastolic, and mean blood pressure. Pulse pressure values were reduced at daytime after tDCS when compared with baseline and SHAM at final moment (Table 5).

**Table 5:** Short-term intervention (10 sessions): 24-hs-ambulatory blood pressure measurements (ABPM) in resistant hypertensive patients at baseline and after 10 sessions of SHAM or tDCS.

		**SHAM**	**TDCS**	***P* Intragroup**	***P* vs. Sham - Final**
**24 h**					
SBP (mmHg)	Baseline	145 ± 10	141 ± 10	0.3441	0.1095
	Final	147 ± 11	148 ± 10	0.4214	
DBP (mmHg)	Baseline	90 ± 10	87 ± 9	0.2987	0.3139
	Final	92 ± 9	89 ± 8	0.1240	
MBP (mmHg)	Baseline	97 ± 9	96 ± 10	0.5474	0.3439
	Final	94 ± 8	95 ± 9	0.6102	
PP (mmHg)	Baseline	57 ± 5	53 ± 6	0.0781	0.0712
	Final	55 ± 7	51 ± 5	0.0702	
**Daytime**					
SBP (mmHg)	Baseline	138 ± 10	139 ± 10	0.7441	0.4889
	Final	139 ± 10	138 ± 11	0.7101	
DBP (mmHg)	Baseline	91 ± 10	90 ± 12	0.8704	0.3141
	Final	92 ± 13	89 ± 8	0.3223	
MBP (mmHg)	Baseline	97 ± 10	98 ± 11	0.3321	0.1911
	Final	96 ± 12	96 ± 10	0.8955	
PP (mmHg)	Baseline	58 ± 5.06	56 ± 7	0.2214	0.0389
	Final	55 ± 9	49 ± 6^†^^*^	0.0425	
**Nighttime**					
SBP (mmHg)	Baseline	132 ± 12	129.50 ± 10.41	0.3322	0.3544
	Final	130 ± 15	127 ± 10	0.2141	
DBP (mmHg)	Baseline	75 ± 10	74 ± 10	0.5574	0.6570
	Final	77 ± 11	74 ± 13	0.1232	
MBP (mmHg)	Baseline	92 ± 9	91 ± 14	0.5512	0.4985
	Final	93 ± 12	93 ± 11	0.7422	
PP (mmHg)	Baseline	55 ± 7	52 ± 5	0.1244	0.2559
	Final	54 ± 6	53 ± 4	0.1011	

*Data are expressed as mean ± standard deviation. Repeated measures ANOVA with Bonferroni post-test. SBP, systolic blood pressure; DBP, diastolic blood pressure; MBP, mean blood pressure; PP, pulse pressure.*

† *P < 0.05 vs. Baseline in the same condition;*

* *P < 0.05 vs. SHAM at Final moment*.

**Figure 2:**
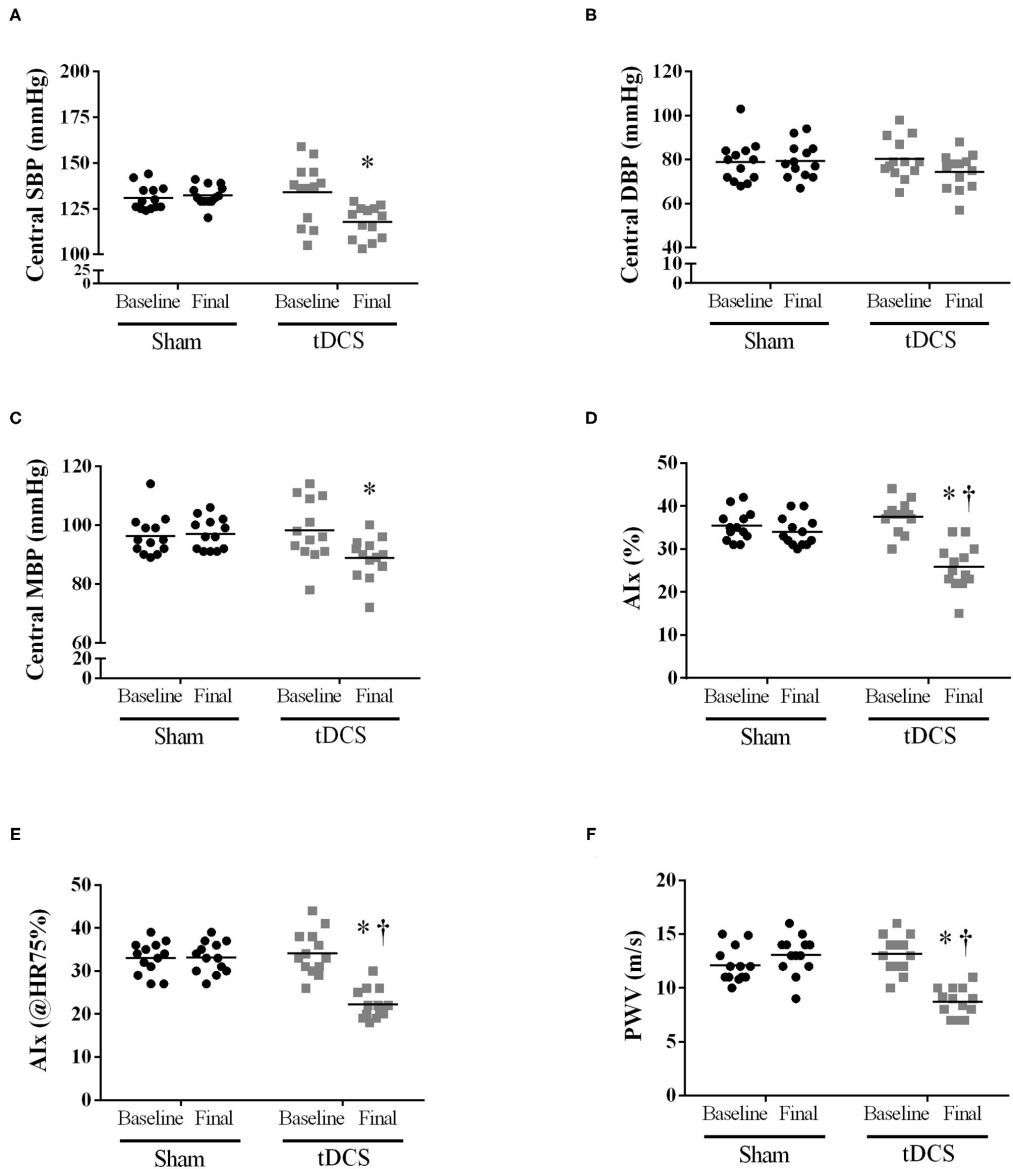
Short-term intervention (10 sessions): Applanation tonometry method to determine whether short-term tDCS (10 sessions) could affect the central blood pressure and pulse waves behavior. **(A)** Central SBP [*P* = 0.0225 vs. SHAM; Interaction – *P* = 0.0016]; **(B)** Central diastolic blood pressure [*p* = 0.2655 vs. SHAM; Interaction – *P* = 0.0106]; **(C)** Central mean blood pressure [*P* = 0.0320 vs. SHAM; Interaction – *P* = 0.0002]; **(D)** Augmentation Index (AIx) expressed in % [*P* = 0.0421 vs. SHAM; Interaction – *P* = 0.0002]; **(E)** AIx normalized for a heart rate of 75 bpm [*P* = 0.0010 vs. SHAM; Interaction – *P* < 0.0001]; **(F)** PWV – pulse wave velocity [*P* = 0.0093 vs. SHAM; Interaction – *P* < 0.0001]. Repeated-measures two-way ANOVA, followed by Bonferroni post-test. *Difference vs. SHAM at final moment; ^†^*P* < 0.05 vs. tDCS baseline in the same condition.

To determine whether 10 sessions of tDCS affects central blood pressure and pulse wave behavior, we used the applanation tonometry method. tDCS reduced central systolic blood pressure [*P* = 0.0225] (Figure 2A), did not change central diastolic blood pressure (Figure 2B), and reduced mean blood pressure [*P* = 0.0320] (Figure 2C) at the final moment in relation to SHAM. Pulse wave amplification indexes (AIx%, Figure 2D) [*P* = 0.0421]), AIx@HR75% [*P* < 0.0001] (Figure 2E), and the pulse wave velocity (PWV, Figure 2F) [*P* = 0.0093] showed a reduction in their values at the final moment in tDCS condition vs. SHAM. Also, tDCS improved these parameters as related to baseline evaluations, as seen to AIx% [*P* = 0.0421], AIx@HR75% [*P* = 0.0010], and PWV [*P* = 0.0093].

**Figure 3:**
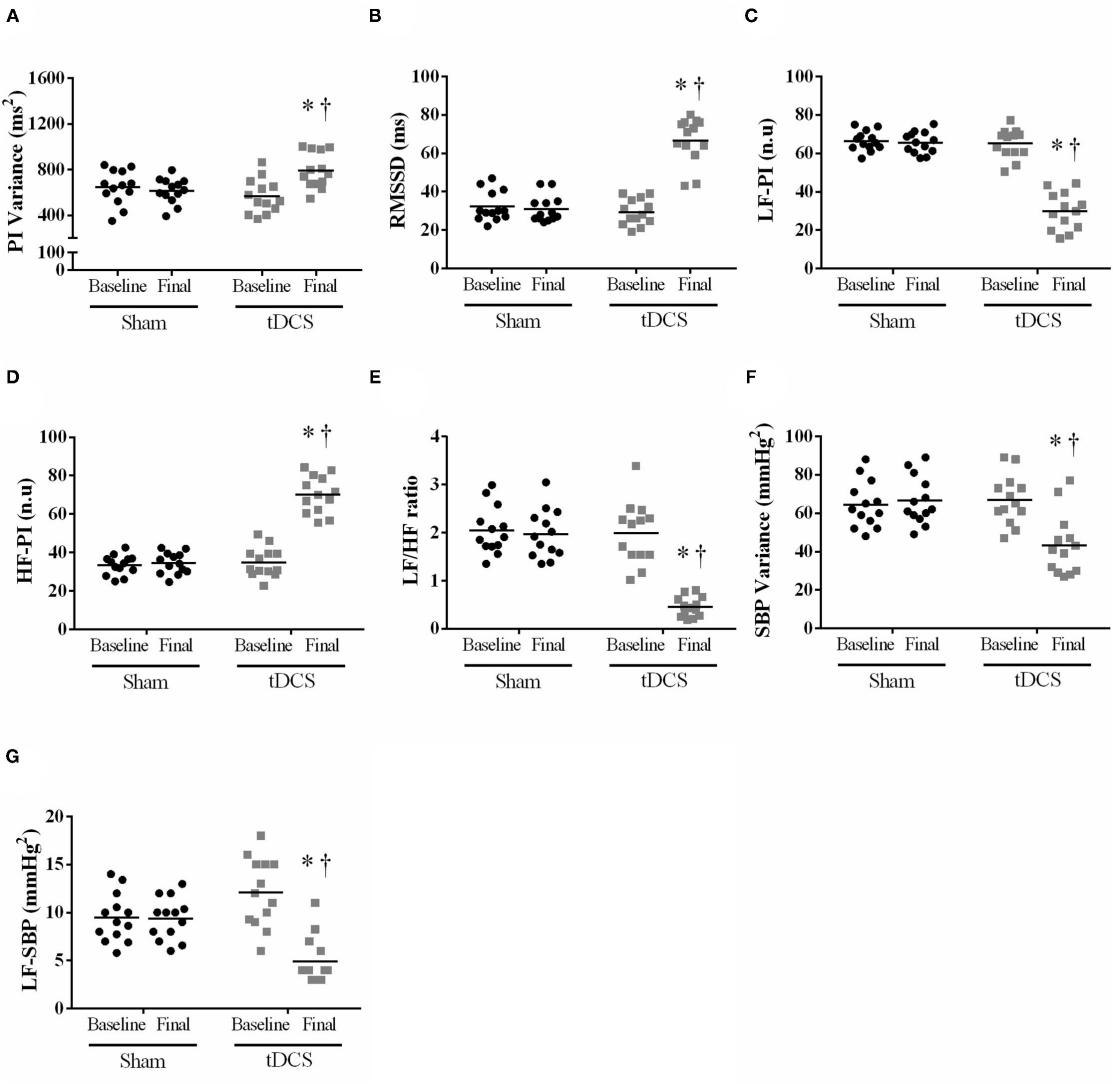
Short-term intervention (10 sessions): tDCS or SHAM effects on pulse interval and systolic blood pressure variability. Repeated measures ANOVA with Bonferroni post-test. **(A)** PI Variance - pulse interval variance [*P* = 0.0006 vs. SHAM; Interaction – *P* = 0.0002]; **(B)** RMSSD - square root of the mean of the square of the differences between adjacent standard RR intervals [*P* = 0.0421 vs. SHAM; Interaction – *P* = 0.0033]; **(C)** LF-PI - low-frequency band of the pulse interval expressed in n.u. [*P* = 0.0002 vs. SHAM; Interaction – *P* = 0.0003]; **(D)** HF-PI - a high-frequency band of the pulse interval expressed in n.u. [*P* < 0.0001 vs. SHAM; Interaction – *P* < 0.0001]; **(E)** LF/HF ratio - autonomic balance [*P* < 0.0001 vs. SHAM; Interaction – *P* < 0.0001]; **(F)** SBP Variance - variance of systolic blood pressure [*P* = 0.0018 vs. SHAM; Interaction – *P* < 0.0001]; **(G)** LF-SBP - low-frequency band of systolic blood pressure [*P* = 0.0012 vs. SHAM; Interaction – *P* < 0.0001]. *Difference vs. SHAM at the final moment; ^†^*P* < 0.05 vs. tDCS baseline.

Given the blood pressure is regulated by the autonomic nervous system, we also investigated whether ten sessions of tDCS can improve cardiac and peripheral autonomic modulation. As can be seen in Figure 3, tDCS increased PI Variance [Figure 3A; *P* = 0.0006], RMSSD [Figure 3B; *P* = 0.0421], reduced LF-PI (n.u.) [Figure 3C; *P* = 0.0002], increased HF-PI (n.u.) [Figure 3D; *P* < 0.0001], as well as decreased LF-HF-ratio [Figure 3E; *P* < 0.0001], SBP Variance [Figure 3F; *P* = 0.0018], and LF-SBP [Figure 3G; *P* = 0.0012] vs. SHAM at the end of experiments.

**Figure 4:**
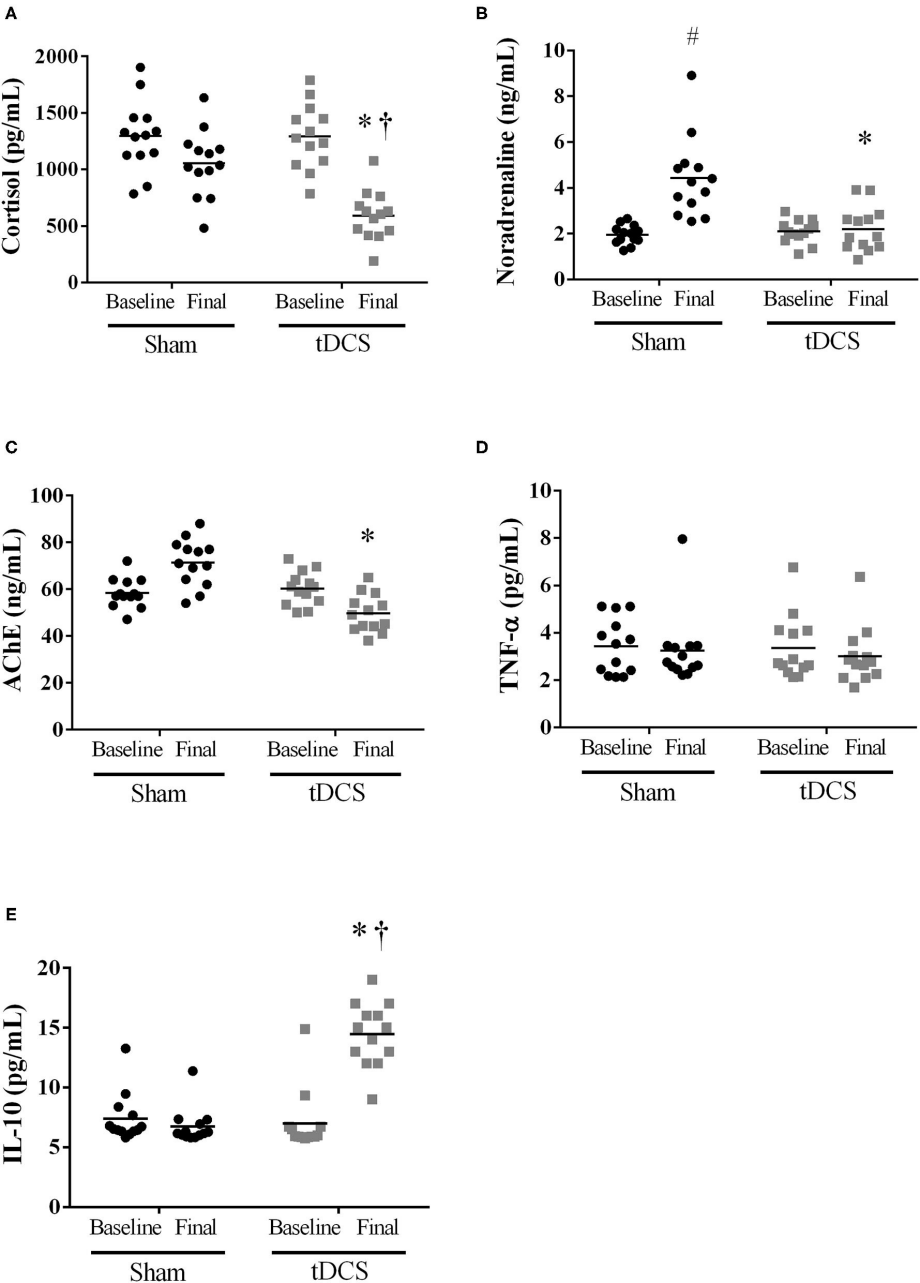
Short-term intervention (10 sessions): Humoral mechanisms associated with BP regulation {**(A)** cortisol [*P* = 0.0056 vs. SHAM; Interaction – *P* = 0.0072]; **(B)** noradrenaline [*P* = 0.0004 vs. SHAM; Interaction – *P* < 0.0001]; **(C)** acetylcholinesterase [*P* < 0.0001 vs. SHAM; Interaction – *P* < 0.0001]} and inflammatory cytokines {**(D)** TNF-α [P=0.5774 vs. SHAM; Interaction – P=0.8195], and **(E)** IL-10 [*P* = 0.0053 vs. SHAM; Interaction – *P* = 0.0062]}. ^#^Difference vs. SHAM baseline; ^†^*P* < 0.05 vs. tDCS baseline; *Difference vs. SHAM at final moment.

Next, we focused on biochemical markers associated with blood pressure regulation, as well as inflammatory cytokines. tDCS condition reduced cortisol levels [vs. SHAM: *P* = 0.0056; vs. Baseline: *P*=0.0033] (Figure 4A); prevented an increase in noradrenaline [vs. SHAM: *P* = 0.0004], as observed in SHAM condition [vs. Baseline SHAM: *P* = 0.0089] (Figure 4B); and acetylcholinesterase measurement was reduced vs. SHAM at final moment [*P* < 0.0001] (Figure 4C). TNF-α values (Figure 4D) [*P* = 0.5774] were no changed by tDCS; however, IL-10 levels (Figure 4E) were increased when compared to SHAM [*P* < 0.0001] at the final, and as compared with baseline evaluation [*P* = 0.0053].

**Figure 5:**
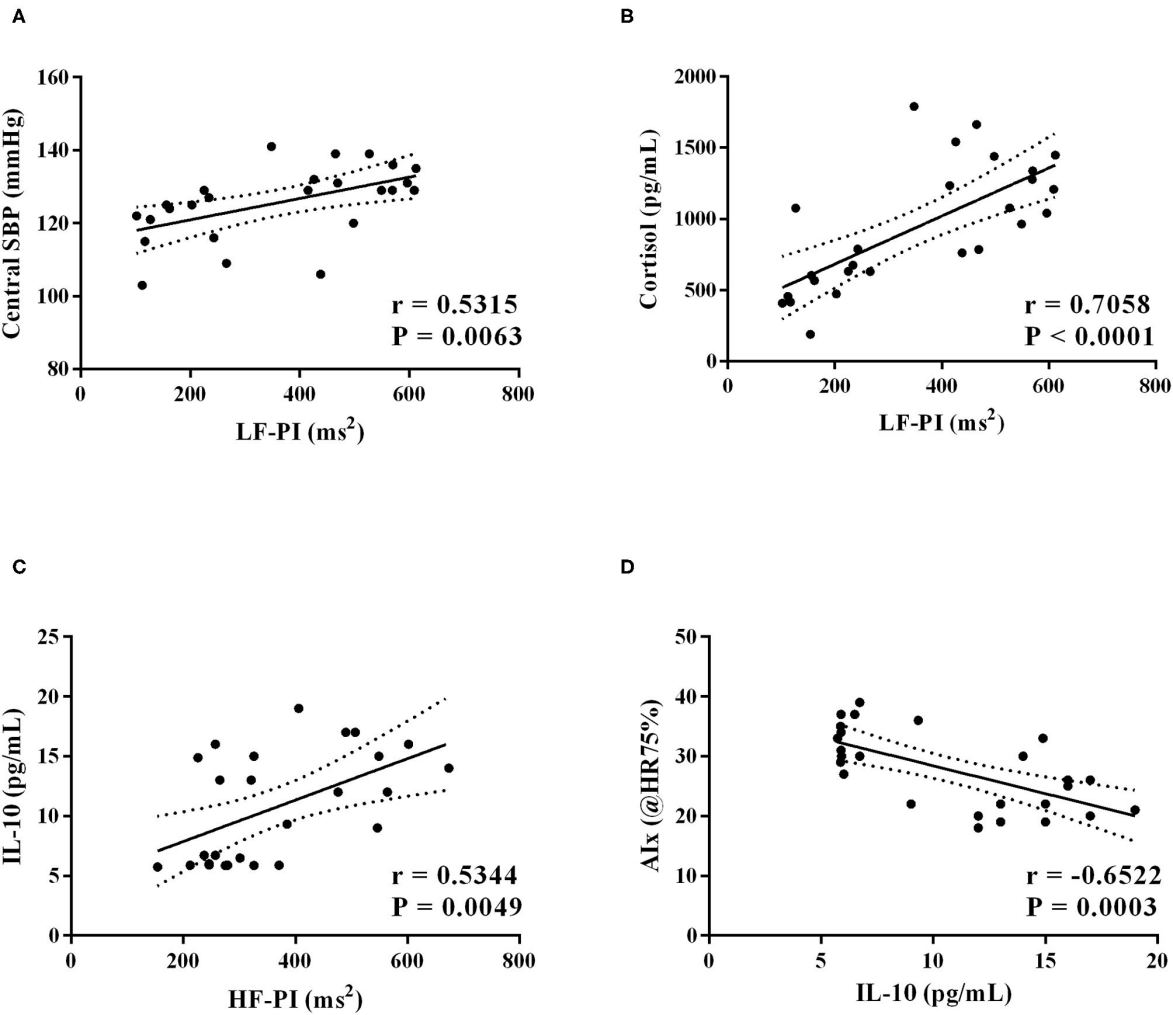
Short-term intervention (10 sessions): Linear regression analysis was carried out and, if the slope of the regression line is significantly larger than 0 with *P* < 0.05, the linear regression (solid line) and its 95% confidence interval (dotted lines) are plotted as well. **(A)** LF-PI and Central SBP [r = 0.5315; *P* =0.0063]; **(B)** LF-PI and Cortisol levels [r = 0.7058; *P* < 0.0001]; **(C)** HF-PI and IL-10 levels [r = 0.5344; *P* = 0.0049]; **(D)** IL-10 and AIx (@HR75%) [r = −0.6522; *P* = 0.0003].

Additionally, we observed positive correlations between: i) LF-PI (ms^2^) band and central SBP [r = 0.5315; *P* = 0.0063] (Figure 5A), ii) LF-PI (ms^2^) band and cortisol levels [r = 0.7058; *P* < 0.0001] (Figure 5B), and iii) HF-PI (ms^2^) band and IL-10 [r = 0.5344; *P* = 0.0049] (Figure 5C). Finally, we found a negative correlation between IL-10 levels and AIx (@HR75%, Figure 5D) [r = −0.6522; *P* = 0.0003].

## Discussion

In the past two decades, experimental and clinical studies of the physiological mechanisms accounting for blood pressure lowering with baroreflex activation (8) and renal denervation in RHT patients (9–11) have grown. We postulated that other forms of non-pharmacologic autonomic modulation, such as tDCS, should be tested for RHT. Herein, we used acute (i.e., one session) and short-term (i.e., ten uninterrupted sessions) tDCS on the primary motor cortex region (M1) to demonstrate these effects since we previously demonstrated that an acute session of tDCS reduced blood pressure and improved cardiovascular autonomic balance in non-resistant hypertensive patients (14).

The present study provides evidence that brain stimulation using tDCS acutely (one session) reduced blood pressure, cardiac output, peripheral vascular resistance, and LF/HF ratio. In addition, short-term tDCS (10 sessions) was not able to reduce blood pressure values. Nevertheless, this short-term intervention reduces LF/HF ratio, central blood pressure, and arterial stiffness in patients with RHT. Another salient finding is that short-term tDCS reduced cortisol and noradrenaline (baseline vs. final) levels, and increases IL-10 plasma concentration. Lastly, we found significant associations between reducing the LF band of pulse interval and improving central systolic blood pressure and cortisol and between increased vagal modulation and higher IL-10 levels.

RHT is considered a disease of complex management, leading researchers to consider alternative treatment approaches (1, 5), and renal denervation has been extensively studied in rigorous design and follow-up (39–43). These studies included patients with early combined systolic/diastolic hypertension and a high probability of response, excluding volunteers with end-stage renal disease, severe hypertension, or isolated systolic hypertension. On the other hand, baroreceptor activation was only tested in experimental procedures or small clinical trials (8). In addition to these invasive interventions aimed at inhibiting the sympathetic activity and improving baroreflex control, our group previously demonstrated that one session of tDCS reduces 24-h ABPM and office BP in patients with controlled hypertension (14), possibly triggered by the reduction of sympathetic modulation. The precise mechanisms underlying the main findings of the present study are not fully understood, but some relevant points should be considered. The central circuitry associated with tDCS cardiovascular and autonomic effects might involve activating the insular cortex and its projections to autonomic brainstem nuclei (44). Notably, a few studies indicated that electrical stimulation applied over the insular cortex could affect autonomic cardiovascular control (45, 46). More specifically, Montenegro et al. (47) showed that tDCS at the left temporal lobe appears to target autonomic control areas, leading to improvements in heart rate variability indexes in healthy subjects. Although the underlying mechanisms are unclear, it is evident that the present results are of significant clinical importance.

In the present study, we demonstrated that tDCS intervention acutely reduces 24 h-ABPM and hemodynamic parameters in RHT. The improvement in cardiovascular autonomic balance triggered by tDCS may have reduced cardiac contractility, reducing stroke volume and cardiac output. However, studies investigating the effects of tDCS on cardiac function are needed.

Short-term (10 sessions) stimulation was not able to reduce blood pressure values in these patients. Several hypotheses can be speculated with regards to the lack of effect in blood pressure after 10 sessions of tDCS, including: tolerability (the development of adaptations at the molecular level similar to the use of drugs and other substances) (48); the sequence of tDCS sessions (there was no consensus about the quantity or interval time between sessions); as well as the use of certain substances such as caffeine, which could have affected the perpetuation of responses (49). On the other hand, central systolic blood pressure, pulse wave velocity, and augmentation index were reduced by short-term tDCS intervention. Increased large-artery stiffness is considered an independent predictor of cardiovascular events and mortality (50). The reduction of pulse wave velocity and improvement of the augmentation index observed in the present study may potentially reflect the prevention of cardiovascular events in RHT. Many studies support the idea that the sympathetic nervous system can modulate arterial stiffness in both healthy and hypertensive subjects, independent of prevailing hemodynamics and vasomotor tone (51, 52).

To further investigate whether tDCS reverses the autonomic imbalance in RHT patients, pulse interval variability and systolic blood pressure variability were assessed. In addition to being associated with vascular remodeling, sympathetic overdrive contributes to arterial hypertension and cardiovascular risk (50). We demonstrated that acute and ten sessions of tDCS increase vagal modulation (represented by RMSSD and HF band indexes) and reduced LF band of pulse interval and systolic blood pressure. These positive changes resulted in reduced autonomic balance (reducing LF/HF ratio) and systolic blood pressure variability. In addition, plasmatic noradrenaline levels were reduced at final evaluation (after ten sessions of tDCS) as compared with baseline. Added to the central circuitry possibly involved in the autonomic control of blood pressure, it is possible that the electrical stimulation in the M1 area directly reduced the release of noradrenaline by the adrenal medulla. This may be a plausible explanation, given that experiments with non-human primates, using trans-neuronal transport of rabies virus, showed that the control of the adrenal medulla is embedded in cortical areas involved in controlling movement (including M1 area), cognition, and affect. Thereby, corticospinal and corticobulbar-spinal pathways may mediate the influence of the motor network on the adrenal medulla (53, 54). On the other hand, SHAM patients displayed increased plasma levels of noradrenaline. We believe that tDCS prevented such an increase.

Studies have strengthened the hypothesis of a direct relationship between the autonomic nervous system and immune responses in recent years. Evidence indicates that cholinergic systems influence inflammatory responses, controlling the release of TNF-α, IL1β, IL-6, and IL-10. Thus, vagal activation appears to suppress inflammation by acetylcholine binding α7-nicotine receptor (26–28). In hypertensive individuals, the long-term elevation of inflammatory protein levels is associated with an accelerated increase of pulse wave velocity, and this accelerated increase is associated with accelerated longitudinal elevation of blood pressure (55). Here, we demonstrate that short-term tDCS intervention increases vagal modulation (HF band), which is associated with increased levels of IL-10. Altogether, we propose that alterations in the autonomic nervous system by tDCS improve pulse wave behavior and contribute to the increase of the anti-inflammatory cytokine IL-10, which, in turn, reduces the production of pro-inflammatory factors, mitigating cardiac and vascular remodeling occurring during RHT. Finally, tDCS reduces cortisol levels in RHT patients. This finding is significant since glucocorticoids may increase the action of angiotensin II, leading to high sympathetic nerve activity, the release of vasopressin, and attenuating the baroreceptor reflex (56).

We recognize some limitations in this study. A small sample size (n = 13) was evaluated in the present study. Although we assessed pharmacological treatment during experiments via pill count, no toxicological evaluation of the adherence was performed. Regarding cardiovascular autonomic studies, the spectral analysis was performed through the pulse interval signals collected by photoplethysmography, being less accurate than the evaluation by the electrocardiogram. Additionally, microneurography's non-use as an additional tool to assess sympathetic nerve activity, and neurotransmitters evaluation involved in the regulation of vascular tone are significant limitations of our study, restricting the conclusions.

## Conclusion

We conclude that acute tDCS can reduce blood pressure in RHT, possibly mediated by autonomic modulation positive changes. After short-term tDCS, resting blood pressure values were not affected. However, autonomic modulation was substantially improved by ten sessions of tDCS. Such changes may have contributed to beneficial alterations in pulse wave behavior and IL-10 concentration. As far as we searched, no previous investigations measured the effects of acute and short-term tDCS sessions on blood pressure and autonomic modulation in RHT subjects as the primary outcome. More extensive clinical trials, biotechnology advances, miniaturizing devices, software developments, and wireless systems may make the technique valuable and easy to use for cardiovascular disorders with autonomic unbalance such as RHT.

## Data Availability Statement

The raw data supporting the conclusions of this article will be made available by the authors, without undue reservation.

## Ethics Statement

The studies involving human participants were reviewed and approved by Ethical in Research Committee of the School of Medical Sciences, University of Campinas (Campinas, Brazil). The patients/participants provided their written informed consent to participate in this study.

## Author Contributions

BR and HM-J contributed to the conception and design of the work. EM, CB, GM, SF-M, JC, CM, and WN contributed to the acquisition, analysis, and interpretation of data for the work. BR, HM-J, AC, and LV drafted the manuscript. LV and AC critically revised the manuscript. All authors gave final approval and agreed to be accountable for all aspects of work and ensuring integrity and accuracy.

## Funding

This study was supported by São Paulo Research Foundation (FAPESP) [grant numbers #2017/21320/4, #2017/24726-1, and #2016/18104-5]. This study was financed in part by the Coordenação de Aperfeiçoamento de Pessoal de Nível Superior – Brasil (CAPES) – Finance Code 001 and National Council for Scientific and Technological Development (CNPq) [grant number #422979/2018-0]. BR and HM-J are fellowships from CNPq (BPQ) [CNPq, grant number #307646/2019-0].

## Conflict of Interest

The authors declare that the research was conducted in the absence of any commercial or financial relationships that could be construed as a potential conflict of interest.

## Publisher's Note

All claims expressed in this article are solely those of the authors and do not necessarily represent those of their affiliated organizations, or those of the publisher, the editors and the reviewers. Any product that may be evaluated in this article, or claim that may be made by its manufacturer, is not guaranteed or endorsed by the publisher.
